# Quantification of Total Phenols and Antioxidants in Coffee Samples of Different Origins and Evaluation of the Effect of Degree of Roasting on Their Levels

**DOI:** 10.3390/molecules27051591

**Published:** 2022-02-28

**Authors:** Lilian Alnsour, Reem Issa, Shady Awwad, Dima Albals, Idrees Al-Momani

**Affiliations:** 1 Department of Pharmaceutical Sciences, Pharmacological and Diagnostic Research Center (PDRC), Faculty of Pharmacy, Al-Ahliyya Amman University, Amman 19328, Jordan; l.alnsour@ammanu.edu.jo; 2 Department of Pharmaceutical Chemistry & Pharmacognosy, Applied Science Private University, Amman 11931, Jordan; sh_awwad@asu.edu.jo; 3 Department of Medicinal Chemistry and Pharmacognosy, Faculty of Pharmacy, Yarmouk University, Irbid 21163, Jordan; dimabals@yu.edu.jo; 4 Department of Chemistry, Faculty of Science, Yarmouk University, Irbid 21163, Jordan; imomani@yu.edu.jo

**Keywords:** coffee, phenols, antioxidant, TEAC, roasting, GAE

## Abstract

Phenolic and antioxidant compounds have received considerable attention due to their beneficial effects on human health. The aim of this study is to determine the content of total phenols and antioxidants in fifty-two coffee samples of different origins, purchased from the Jordanian local market, and investigate the effect of the degree of roasting on the levels of these compounds. The coffee samples were extracted using the hot water extraction method, while Folin–Ciocalteu (FC) and 1,1-diphenyl-2-picrylhydrazyl (DPPH) assay methods were used to analyze these compounds. The results showed that the highest content of total phenol (16.55 mg/g equivalent to GAE) was found in the medium roasted coffee, and the highest content of antioxidants (1.07 mg/g equivalent to TEAC) content was found in the green coffee. Only light and medium roasted coffee showed a significant correlation (*p* < 0.05, R^2^ > 0.95) between the average of total phenolic and antioxidant content. A negative correlation between the antioxidant content and the degree of roasting (*p* < 0.05, R^2^ > 0.95) were shown, while it did not correlate with phenolic contents. Previously, a positive correlation between antioxidant and chlorogenic acids content was observed, with no correlation between the origin of coffee samples nor heavy metal content, which was previously determined for the same coffee samples. These findings suggest that the antioxidant content for coffee extracts is largely determined by its chlorogenic acid content, rather than the coffee origin or total phenolic and heavy metals content.

## 1. Introduction

Coffee is one of the most popular beverages in the world. It was introduced to the New World in the mid-17th century, although its history dates to the 15th century when coffee plants were supposedly cultivated in Southern Arabia and taken originally from Ethiopia. It was not until the 1950s that instant coffee was produced. Nowadays, Brazil is the top coffee-producing country in the world, followed by Vietnam and Colombia [1].

There are over 120 species of Coffea plant, with *Coffea arabica* and *Coffea canephora* (also known as “Robusta”) being the most popular commercially. The former contributes to 70% of the world’s coffee consumption. The latter contains more caffeine and lower lipid content, which is why it tastes more bitter [2]. It is also cheaper to produce compared with *Coffea arabica* [3]. Green coffee beans are the seeds from the coffee tree fruits. The same species of coffee can be cultivated differently to produce a wide variety of coffee beans having different flavors and aromas, depending on the soil, climate, and altitude of their growing areas, which means that coffee is affected by its geographical origin [4].

Roasting is also an important determinant of taste and aroma in brewed coffee. Green coffee beans are heated at 200–240° for 10–15 min, depending on the degree of roasting required. This may considerably alter their chemical composition; lowering the sugars, water, and chlorogenic acids while forming new compounds, such as melanoidins due to the Maillard reaction [5]. In a study by Mayer and others [6], it was shown that the concentration of certain compounds in roasted coffee beans was greatly affected by the degree of roast. For example, Colombian and Kenyan coffees have increasing amounts of phenolic compounds, such as guaiacol, with an increased roasting degree.

The quality of coffee can be affected by levels of chemical fertilizers and pesticides used in soil, which contribute to heavy metal contamination. The preparation method and degree of roasting also affect its final heavy metal composition. Albals et al. [7] determined the heavy metal content in different green and roasted coffee samples consumed in Jordan, taken from five origins: Brazil, Ethiopia, Kenya, Colombia, and India. According to the results, there was a significant difference in the levels of Zn, Cr, and Co in green and roasted coffee beans. All levels were below the tolerable upper limit of daily intake (TULD) of metals determined by the World Health Organization (WHO) and thus were safe for consumption.

Coffee is known to be an essential source of antioxidants due to the presence of alkaloids, flavonoids, and phenolic compounds. Consumption of coffee is therefore attributed to improving health [4]. Moreover, it is the main antioxidant found in the diets of Americans, Japanese, Danish, and Brazilians [8].

Phenolic compounds are widely abundant in fruits, vegetables, dry legumes, chocolate, and beverages like coffee, tea, and cocoa [9]. Polyphenols have been shown to have antioxidant effects, which are beneficial to the heart and can protect against oxidative stress that is directly correlated with degenerative diseases, diabetes mellitus, and cancer [10]. Other evidence suggests their anti-inflammatory, antiviral, and antibacterial activity [11]. For instance, green coffee beans have been reported to contain chlorogenic and caffeic acids as the main phenols. These compounds possess antimutagenic and antioxidative effects [8]. Other studies suggest its role in neurodegenerative diseases, such as ischemic strokes and lowering blood pressure in rats [12].

A study by Masek et al. [8] on five different Ethiopian coffee brands has demonstrated a significant total phenolic content and antioxidant activities, which showed that Ethiopian coffee might be used in preventing and curing several degenerative diseases. Another study by Sentkowska et al. [5] was carried out to determine the antioxidant capacity of different green coffee extracts. It has been reported that Robusta green coffee from Laos had the highest antioxidant capacity due to the high concentration of chlorogenic acids. Duarte et al. [13] concluded that roasting is inversely proportional to the polyphenol and antioxidant activity, where light brewed coffee had the highest antioxidant capacity, while dark roasted coffee had the lowest.

While many studies were conducted in Jordan to study the total phenolic content and antioxidant capacity of various plant species [14], only a few of them were carried out on coffee. A previous study by Kandah et al. [14] analyzed total phenolic content and antioxidant activity using methyl linoleate (MeLo) assay for nine samples of green and roasted coffee beans obtained from the Jordanian market. It demonstrated that extraction time, temperature, and particle size were important variables that affected total phenolic content. Another study compared the antioxidant activity of roasted barley and roasted dates with that of two different roasted coffee samples and green coffee samples (Saudi and Colombian origins) using ABTS and DPPH assays. Results indicated that the highest antioxidant activity was observed for Saudi roasted coffee, followed by Colombian roasted coffee, roasted barley, Colombian green coffee, and roasted dates [15].

To the best of our knowledge, this is the first extensive study conducted in Jordan on 52 different coffee samples with the aim to evaluate the effect of roasting and geographical origin on the antioxidant and total phenolic content of coffee beans available in the Jordanian market, using 1,1-diphenyl-2- picrylhydrazyl (DPPH) and Folin–Ciocalteu (FC) assays, respectively, for this purpose. All samples were prepared by water extraction similar to the way normally used when preparing the beverage, to investigate the individual’s intake of antioxidant phenols by consuming coffee brew. The secondary aim was to find a correlation (if any) between the antioxidant activity and total phenolic content of different types of coffee beans, with their caffeine, chlorogenic acids, and heavy metal content, the latter that were previously determined by our research group (Albals et al.) (Awwad et al.) [7,16].

## 2. Results

### 2.1. Total Polyphenols Content

**Figure 1:**
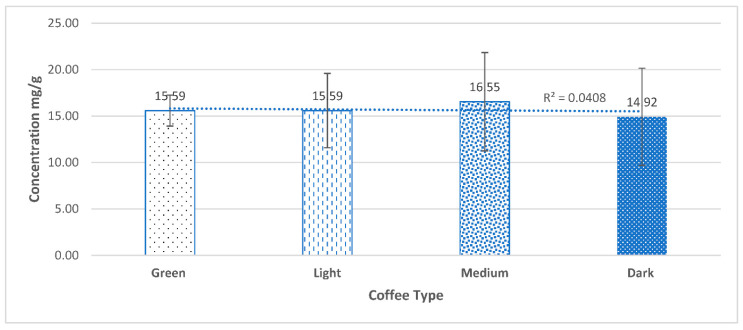
The average total phenolic content (mg/g GAE) for each roasting degree with (different geographical origin). No correlation was found between roasting degree and total phenolic content. (R^2^ = 0.041). Results are statistically insignificant with a *p*-value > 0.05.

The average total phenolic content, expressed as milligrams of gallic acid equivalents per gram of dry coffee extract (GAE mg/g), was determined for 52 *Coffea arabica* samples of different origins and roasting degrees, as shown in Supplementary Table S1. These samples have shown variations in total phenolic content ranging from 14.92 mg/g to 16.55 mg/g (Figure 1), reported for dark roasted coffee and medium roasted coffee, respectively. Except for green coffee beans, the variety from Kenya showed to have the lowest total phenolic content, while the variety from Colombia showed to have the highest total phenolic content (Table 1). For green coffee beans, the variety from Kenya showed to have the highest total phenolic content, which was decreasing in its content by the roasting process in varying amounts. While the variety from Colombia showed the opposite behavior upon roasting, as its content of phenols tends to increase by increasing the roasting degree.

### 2.2. Antioxidant Activity of Coffee Samples

**Figure 2:**
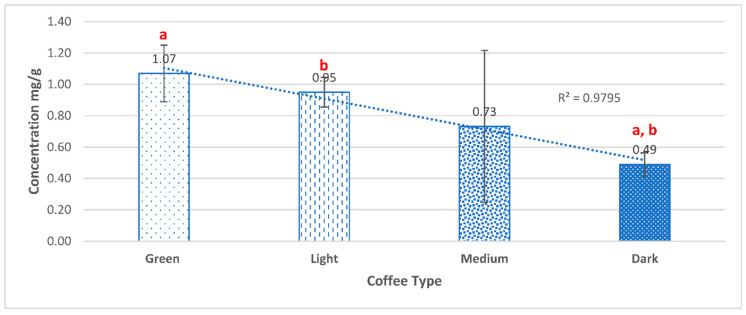
The average antioxidant activity (mg/g TEAC) for each roasting degree (different geographical origin) A negative correlation was found between roasting degree and TEAC mg/g (R^2^ = 0.98). Bars labeled ^a,b^ are statistically significant with a *p*-value < 0.05.

The average antioxidant activity—expressed as milligrams of Trolox equivalents antioxidant capacity per gram dry coffee extract (TEAC mg/g)—was determined for 52 *Coffea arabica* samples of different origins and roasting degrees, as shown in Supplementary Table S1. The values ranged from 0.49 mg/g to 1.07 mg/g, reported for dark roasted coffee and green coffee, respectively (Figure 2). The antioxidant activity was correlated with the roasting degree of coffee beans. Although the content of phenols showed a consistent pattern in terms of the geographical origin of coffee beans, no correlation was found for the antioxidant activity with the origin (Table 1). Only light and medium roasted coffee showed a significant correlation between the average total phenolic content and the average antioxidant content.

### 2.3. Comparison of the Content of Total Phenol, Antioxidant, and Heavy Metal in Selected Coffee Samples

**Figure 3:**
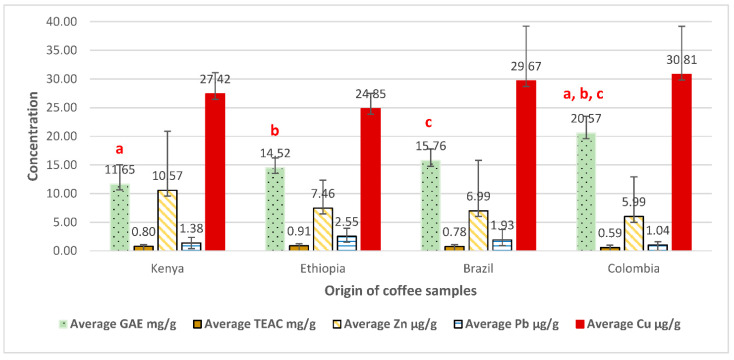
Comparison of coffee samples from different origins (regardless of their roasting degree) in terms of average GAE in mg/g, average TEAC in mg/g, and average Zn, Pb, and Cu contents in µg/g. (Data for heavy metal content taken from Albals et al. [7]). Bars labeled ^a,b,c^ are statistically significant with a *p*-value < 0.05.

**Figure 4:**
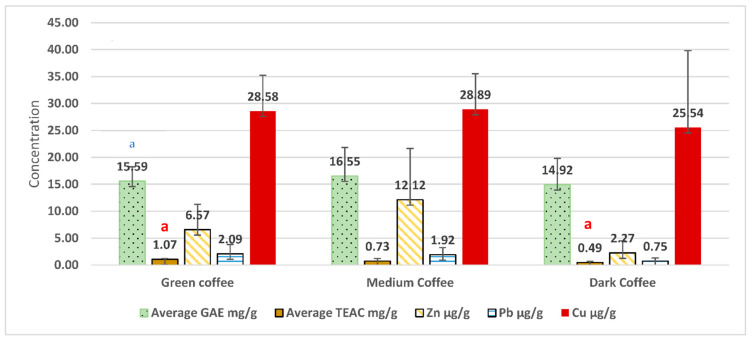
Comparison of coffee samples of three different roasting degrees (regardless of their origin) in terms of average GAE in mg/g, average TEAC in mg/g, and average Zn, Pb, and Cu contents in µg/g. (Data for heavy metal content was taken from Albals et al. [7]). Bars labeled ^a^ are statistically significant with a *p*-value < 0.05.

Figure 3 and Figure 4 show a comparison of coffee samples of different geographical origins (regardless of their roasting degree) and roasting degrees (regardless of their origin) in terms of average GAE in mg/g, average TEAC in mg/g, and average Zn, Pb, and Cu contents in µg/g (Data for heavy metal content was taken from Albals et al. [7]).

## 3. Discussion

The roasting degree and geographical origins are key factors affecting both the total phenolic content and the antioxidant activity of the coffee beans [17]. Therefore, this study focused on evaluating the effect of these variables on the antioxidant and phenolic compound contents in coffee samples available in the Jordanian market. In addition, this study aimed to explore the individual intake of phenols and antioxidants from coffee beverages commonly used among Jordanian citizens, known as “Turkish coffee”. Since antioxidant compounds provide health benefits, coffee as a beverage is claimed to be of great interest for individuals who are trying to increase their intake of these nutrients [18]. Consequently, such findings should reveal the actual individual intake of these nutrients that would influence their health status.

The findings of the current study are in good agreement with the previous study by Król et al. [17], which showed that the highest content of total polyphenolic compounds was determined in coffee samples roasted in light and medium roasting conditions, and was better for preserving these nutrients.

A previous study by Bobková et al. [19] showed a correlation between phenolic content and antioxidant activity. On the contrary, the current findings showed that no correlation was found between phenolic content, antioxidant activity, or geographical origin. Medium roasted beans appeared to contain higher average polyphenols than the green beans, where the variety from Colombia showed to have the highest phenolic content among the roasted samples (regardless of the roasting degree), but with varied antioxidant activity.

As expected, statistical analyses of these data revealed a significant influence (*p* < 0.05. R^2^ = 0.98) of the roasting degree on the antioxidant activity, which was decreasing with the increase in the degree of roasting. Cho et al. [20] studied the influence of roasting conditions on the antioxidant characteristics of Colombian coffee of the species *Coffea arabica* beans. They found that light-roasted coffee beans have the highest antioxidant activity, and an approximately 40–80% loss of antioxidant activity was observed after further roasting. In addition, they also detected significantly higher antioxidant activity as compared to unroasted beans, suggesting the formation of Maillard reaction products and the release of bound polyphenols from plant cells. These data suggested the formation of new phenolic compounds, other than the ones detected in the variety from Colombia, but with no effect on their antioxidant content.

Green coffee beans showed the highest content of phenols in the species from Kenya. The latter also showed the highest antioxidant activity compared to the other studied samples. In fact, different varieties of coffee samples showed a low and narrow range of phenolic content compared to the previous studies [21,22]. This could be explained by the fact that phenolic compounds are often more soluble in alcohol extracts compared to water, which was used in this study.

A previous study by Górnaś et al. [23], which investigated the contribution of phenolic acids isolated from green and roasted boiled-type coffee brews to total coffee antioxidant capacity, showed that the antioxidant effect can be poorly correlated with polyphenols’ concentration when the DPPH assay method is used, which agrees with the findings of this study. The antioxidant activity changes in extracts from green, light, medium, and dark roasted coffee are negatively influenced by the intensity of the heating process and seem to be much more dependent on the roasting degree than on the geographical origin of coffee beans. Similarly, Bilge [24] conducted a study investigating the effects of geographical origin, roasting degree, particle size, and brewing method on the physicochemical and spectral properties of Arabica coffee. It showed that roasting degree and brewing method—compared with other parameters—were the most discriminating factors based on UV and fluorescence spectra of coffee brew samples, respectively. On the contrary, Muzykiewicz-Szymańska et al. [25] studied the effect of brewing process parameters on antioxidant activity in infusions of roasted and unroasted Arabica coffee beans originating from different countries. They concluded that the phenolic compound content in infusions prepared using different techniques depended on the roasting process, the bean’s origin, as well as the brewing technique.

Based on the results from the study by Albals, et al. [7] which investigated the heavy metals contents for the same coffee samples that were analyzed in this study, the data showed that there was no clear correlation between the content of phenols, antioxidant compounds, and heavy metals content. Therefore, these findings suggest that roasting degree would affect the antioxidant activity, regardless of the geographical origin or heavy metal content. Nevertheless, the geographical origin had shown an impact only on the total phenolic content, with no effect on any of the other measured variables.

Results from a study—that was recently published by our research group—which determined the chlorogenic acids (CGAs) and caffeine content for the same coffee samples, have shown that the highest content of caffeine was found in the medium roasted coffee (203.63 mg/L), and the highest content of CGA was found in the green coffee (543.23 mg/L). The results demonstrated a negative correlation between the CGA levels with the degree of roasting, while it showed a positive correlation between the caffeine levels with the degree of roasting before it starts to decline in the dark roasted coffee [16].

Comparing these results with the current study, it can be concluded that the coffee samples with the highest CGA content have also shown the highest antioxidant activity, which suggests that CGAs alone rather than total phenolic content contribute to the antioxidant activity of coffee. Furthermore, the geographical origin did not seem to affect the content of either CGA or caffeine, as it did not affect the total phenolic content and antioxidant activity determined in this study [16].

This study showed the need to perform more research using different assays to investigate the relationship between the antioxidant activity with the total phenolic content.

## 4. Methods and Materials

### 4.1. Chemicals and Standards

For the determination of the total phenolic content, the FC reagent (2 N, Sigma-Aldrich, Schaffhausen, Switzerland), gallic acid (GA) (99% purity, Sigma), and anhydrous sodium carbonate (99% purity, Sigma) were used. For the determination of the antioxidant activity, DPPH (Aldrich, Darmstadt, Germany), Trolox (Sigma-Aldrich, Switzerland), and methanol (for HPLC > 99.9%, Sigma-Aldrich, St. Quentin Fallavier, France) were employed.

For this study, fifty-two samples of ground coffee beans (*Coffea arabica),* including green (11 samples), light-roasted (14 samples), medium roasted (11 samples), and dark roasted (16 samples), from different origins were purchased from different grocery stores across Jordan (Amman, Irbid) in 2019 and stored in the freezer.

### 4.2. Sample Preparation and Extraction

The coffee samples were freshly extracted according to the extraction procedure described by Perez Hernandez et al. [26], with few modifications. The coffee samples were extracted with hot water at 75–80 °C at a 1/100 coffee-to-solvent ratio, where 1 g of coffee was extracted with 100 mL of water. Then, ultrasonication was performed for 5 min to homogenize the solutions using an ultrasonic bath (OVAN). Afterwards, the samples were centrifuged for 15 min at 7900× *g* using an (MPW-260R) centrifuge system. Next, all coffee solutions were filtered with Whatman No. 2 filter paper. Finally, the coffee extracts were stored at a temperature of −20 °C until the day of analysis (May–August 2020).

### 4.3. Determination of Total Phenolic Content

Total phenolic content was measured using the FC method by Singleton et al. [27]. A stock solution of 10 mg/mL of extract in water was prepared for each sample. Three different concentrations were made for each extract by serial dilution; 5 mg/mL, 2.5 mg/mL and 1.25 mg/mL. An aliquot of 80 µL of each aqueous solution of extract was added to 400 µL of dilute Folin–Ciocalteu (1:10) reagent in a test tube. Then 320 µL of 7.5% sodium carbonate solution was added. The solution was covered with aluminum foil and incubated in a water bath at 45 °C for 30 min. The absorbance was recorded at 765 nm using a UV-Vis spectrophotometer against the blank solution (water only). The measurement was compared to a calibration curve prepared with GA solution at a concentration range from (0–0.12 mg/mL), and the total phenolic content was expressed as GAE mg/g, using the standard curve equation:

y = 7.515x + 0.0308, R^2^ = 0.9927

where y is the absorbance at 765 nm and x is the total phenolic content in the different extracts expressed in mg/mL.

### 4.4. Determination of Antioxidant Capacity

Aliquots of 2 mL of each plant extract were dried in the oven overnight at a temperature of 50 °C to dry all the water. An amount of 2 mL of methanol was added to each dried sample and vortexed for 3 min to allow a homogenous stock solution of concentration 10 mg/mL. Three different concentrations were made for each extract by serial dilution; 5 mg/mL, 2.5 mg/mL and 1.25 mg/mL. A solution of 0.1 mM DPPH in methanol was prepared fresh for the assay. Then, 300 µL of the sample was added to 100 µL of DPPH solution, shaken, and incubated for 30 min in the dark at room temperature. Absorbance was monitored at 517 nm using a UV-Vis spectrophotometer. The reaction mixture containing control and reference standard (300 µL methanol and 100 µL DPPH solution) were also measured. The measurement was compared to a calibration curve prepared with Trolox solution at a concentration range from (0–50 µM). The antioxidant capacity was expressed as TEAC mg/g, using the standard curve equation:

y = −0.024x + 1.36, R^2^ = 0.992

where y is the DPPH absorbance at 517 nm and x is the Trolox concentration in the different extracts expressed in µM, which is then expressed as mg TEAC/g of dry extract.

### 4.5. Statistical Analysis

All measurements were performed in triplicates and results were reported as mean ± standard deviation. The results were analyzed statistically using one-way analysis of variance (ANOVA) on Microsoft Excel with its data analysis add-ins. The mean values of GAE mg/g were compared with TEAC mg/g to assess the existence of statistical significance using measurements of *p*-value and squared correlation coefficients (R^2^). The level of significance was set to 0.05 and 0.95, respectively.

## Figures and Tables

**Table 1:** Comparison of different roasting degrees for coffee samples from different geographical origins according to their total phenolic and antioxidant activity (GAE mg/g and TEAC mg/g, respectively).

Geographical Origin	GAE mg/g ± SD	TEAC mg/g ± SD
Green Coffee		
Kenya	17.25 ± 0.14	1.29 ± 0.04
Ethiopia	14.55 ± 0.10	1.13 ± 0.11
Brazil	13.82 ± 0.13	0.97 ± 0.14
Colombia	16.72 ± 0.05	0.88 ± 0.08
Average	15.59 ± 1.65	1.07 ± 0.18
Light Coffee		
Kenya	9.84 ± 0.02	0.84 ± 0.05
Ethiopia	16.35 ± 0.05	0.96 ± 0.03
Brazil	17.11 ± 0.05	0.94 ± 0.07
Colombia	19.05 ± 0.13	1.07 ± 0.14
Average	15.59 ± 4.00 *	0.95 ± 0.09 *
Medium Coffee		
Kenya	12.31 ± 0.02	0.65 ± 0.12
Ethiopia	14.93 ± 0.14	1.13 ± 0.01
Brazil	14.66 ± 0.05	1.07 ± 0.07
Colombia	24.28 ± 0.11	0.08 ± 0.02
Average *	16.55 ± 5.29 **	0.73 ± 0.48 **
Dark Coffee		
Kenya	9.44 ± 0.11	0.60 ± 0.10
Ethiopia	12.24 ± 0.14	0.43 ± 0.10
Brazil	16.60 ± 0.14	0.47 ± 0.17
Colombia	21.41 ± 0.20	0.46 ± 0.15
Average	14.92 ± 5.23	0.49 ± 0.07

*, ** Results are statistically significant with *p*-value < 0.05, R^2^ > 0.95.

## Data Availability

Not applicable.

## Supplementary Materials

The following supporting information can be downloaded online, Table S1: The distribution of Coffea arabica samples (52) obtained from the Jordanian market according to their origin and roasting degree. Roasting temperatures for light roasted = 155–165 °C, for medium roasted = 175–185 °C, and for dark roasted = 205–215 °C. Table S2: The Average concentration (mg/g), SD, SE, Min, Max, and confidence interval for GAE in coffee beans obtained from the Jordanian market. Table S3: The Average concentration (mg/g), SD, SE, Min, Max, and confidence interval for TEAC in coffee beans obtained from the Jordanian market.

## Sample Availability

Samples of the standard compounds are available from the authors.
