# Supplementary material for: Quantification of Total Phenols and Antioxidants in Coffee Samples of Different Origins and Evaluation of the Effect of Degree of Roasting on Their Levels
Source: Molecules. 2022 Feb 28;27(5):1591. doi: 10.3390/molecules27051591 (PMC8912094; doi:10.3390/molecules27051591)
Supplement: Supplementary file 1 [file molecules-27-01591-s001.zip › molecules-1561451-supplementary.pdf]

molecules

Article

Quantification of Total Phenols and Antioxidants in Coffee Samples of Different Origins and Evaluation of the Effect of Degree of Roasting on their Levels

Lilian Alnsour [1], Reem Issa [1*], Shady Awwad [2], Dima Albals [3], and Idrees F. Al-Momani [4]

[1] Department of Pharmaceutical Sciences, Pharmacological and Diagnostic Research Center (PDRC), Faculty of Pharmacy, Al-Ahliyya Amman University, Amman, Jordan
[2] Department of Pharmaceutical Chemistry & Pharmacognosy, Applied Science Private University, Amman, Jordan
[3] Department of Medicinal Chemistry and Pharmacognosy, Faculty of Pharmacy, Yarmouk University, Irbid, Jordan
[4] Department of Chemistry, Faculty of Science, Yarmouk University, Irbid, Jordan
*Corresponding author: r.issa@ammanu.edu.jo

Table S1: The distribution of *Coffea arabica* samples (52) obtained from the Jordanian market according to their origin and roasting degree. Roasting temperatures for light roasted =155-165 °C, for medium roasted= 175-185°C, and for dark roasted= 205-215°C.

Sample Origin	Number of samples	Degree of roasting/type	Concentration of extract
Kenya	2*	Green	5mg/mL
	2*	Light	5mg/mL
	2*	Medium roasted	5mg/mL
	2*	Dark	5mg/mL
	1	Over roasted***	5mg/mL
Saudi Arabia**	1	Green	5mg/mL
Ethiopia	3*	Green	5mg/mL
	1	Light	5mg/mL
	1	Medium roasted	5mg/mL
	1	Dark	5mg/mL
Brazil	2*	Green	5mg/mL
	4*	Light	5mg/mL
	4*	Medium roasted	5mg/mL
	4*	Dark	5mg/mL

	**1**	**Over roasted*** **	**5mg/mL**
**Colombia**	**3***	**Green**	**5mg/mL**
	**4***	**Light**	**5mg/mL**
	**4***	**Medium roasted**	**5mg/mL**
	**5***	**Dark**	**5mg/mL**
	**1**	**Over roasted*** **	**5mg/mL**
**India****	**3***	**Light**	**5mg/mL**
	**1**	**Over roasted*** **	**5mg/mL**

Samples of the same origin and roasting degree that were purchased from different stores across Jordan were blended to homogenize results. **Indian and Saudi coffee samples were excluded from the graphs due to lack of samples with varying roasting degrees available.Results for over-roasted samples were combined with those of dark roasted coffee samples for the same origin.**

Table S2: The Average concentration (mg/g), SD, SE, Min, Max, and confidence interval for GAE in coffee beans obtained from the Jordanian market.

Descriptives

average_GAE

	N	Mean	Std. Deviation	Std. Error	95% Confidence Interval for Mean Lower Bound	95% Confidence Interval for Mean Upper Bound	Minimum	Maximum
"Green"	4	15.5850	1.65800	0.82900	12.9468	18.2232	13.82	17.25
"Light"	4	15.5875	3.99675	1.99838	9.2278	21.9472	9.84	19.05
"Medium"	4	16.5450	5.28920	2.64460	8.1287	24.9613	12.31	24.28
"Dark"	4	14.9225	5.23308	2.61654	6.5955	23.2495	9.44	21.41
Total	16	15.6600	3.89532	0.97383	13.5843	17.7357	9.44	24.28

Table S3: The Average concentration (mg/g), SD, SE, Min, Max, and confidence interval for TEAC in coffee beans obtained from the Jordanian market.

Descriptives

average_TEAC

	N	Mean	Std. Deviation	Std. Error	95% Confidence Interval for Mean Lower Bound	95% Confidence Interval for Mean Upper Bound	Minimum	Maximum
"Green"	4	1.0675	0.18081	0.09040	0.7798	1.3552	0.88	1.29
"Light"	4	0.9525	0.09430	0.04715	0.8025	1.1025	0.84	1.07
"Medium"	4	0.7325	0.48459	0.24229	-0.0386	1.5036	0.08	1.13

"Dark"	4	0.4900	0.07528	0.03764	0.3702	0.6098	0.43	0.60
Total	16	0.8106	0.32927	0.08232	0.6352	0.9861	0.08	1.29
